# The prevention of rectovaginal fistula after rectal cancer surgery by packing with laparoscopic dislocated fat flap containing ovarian vascular pedicle anterior to the anastomotic stoma: a parallel group randomized controlled trial protocol

**DOI:** 10.1186/s13063-023-07721-2

**Published:** 2024-01-18

**Authors:** Dao-xiong Ye, Sheng-hui Huang, Yu Lin, Ying-Huang, Pan Chi

**Affiliations:** Department of Colorectal Surgery, Fujian Medical University Union Hospital, 29 Xinquan Road, Fuzhou, 350001 Fujian Province China

**Keywords:** Rectovaginal fistula, Fat flap containing ovarian vascular pedicle, Ovarian function failure, Post-menopause, Rectal cancer, Randomized controlled trial

## Abstract

**Background:**

Rectovaginal fistula (RVF) is an abnormal channel formed by epithelial tissue between the anterior wall of the rectum and the posterior wall of the vagina, which manifests as vaginal gassing and defecation. It is one of the common complications of female pelvic surgeries. With the increased number of proctectomies for rectal cancer, the number of postoperative rectovaginal fistulas also increases. Once RVF occurs, the failure rate is still high with various treatments available. RVF causes great suffering to women and is still a major problem in treatment. Therefore, it is significant for female rectal cancer patients to prevent RVF after rectal cancer surgery. In this study, we introduce a new method to prevent RVF during rectal cancer radical operation.

**Methods:**

In this randomized controlled trial (RCT), all operations are performed according to the principle of total mesorectal excision (TME) radical resection in rectal cancer surgery. All eligible participants will be divided into two groups: the experimental group and the control group. Experimental group: the anterior rectal wall of about 1 cm distal to the anastomosis was dislocated. Before the anastomosis of the rectal end, a fat flap (usually left side) containing the ovarian vascular pedicle was dislocated, measured by 10–15 cm in length and 2 cm in width. The fat flap containing the ovarian vascular pedicle was packed and fixed anterior to the anastomotic stoma with fibrin glue. Control group: surgery will be carried out in accordance with the TME principle. Participants will be compared on several variables, including the incidence of RVF after operation (primary outcomes), the occurrence time of postoperative RVF, the occurrence time of RVF after stoma closure, and other postoperative complications, such as anastomotic leakage, chylous leakage, and intestinal obstruction (secondary outcomes). The follow-up data collection will be conducted according to the follow-up time point, and the baseline data will also be collected for follow-up analysis. By comparing the incidence of rectovaginal leakage between the experimental group and the control group, we aim to explore the feasibility of this method for the prevention of postoperative RVF.

**Discussion:**

This RCT will explore the feasibility of packing with a laparoscopic dislocated fat flap containing an ovarian vascular pedicle anterior to the anastomotic stoma after rectal cancer surgery to prevent RVF.

**Trial registration:**

Chinese Clinical Trial Registry (ChiCTR) registration ChiCTR2000031449. Registered on June 26, 2019. All items of the WHO Trial registration data set can be found within the protocol.

**Supplementary Information:**

The online version contains supplementary material available at 10.1186/s13063-023-07721-2.

## Introduction

### Background and rationale {6a}

Rectovaginal fistula (RVF) is an abnormal channel formed by epithelial tissue between the anterior wall of the rectum and the posterior wall of the vagina, which manifests as vaginal gassing and defecation. It is one of the common complications of female pelvic surgeries. With the increased number of proctectomies for rectal cancer, the number of postoperative RVF also increases with the incidence reported to be 0.9–9.9% in most literatures [1, 2]. Once RVF occurs, the failure rate is still high with various treatments available [3]. Most of the existing studies focus on the treatment instead of prevention for RVF. Recent researches on postoperative RVF of low-middle rectal cancer have reported that intraoperative measures can effectively reduce the incidence of RVF in terms of surgical techniques [4], such as pushing and pulling the uterine and vaginal walls upward and outward which can make a certain tension with the anterior rectal wall; control the frequency of electric knife or ultrasonic knife; examining the vaginal wall via pelvis and vagina prior anastomat triggering to avoid being clamped; and blocking the vaginal wall with pressure intestinal spatula.

Researchers [5, 6] had tried to trancanal pulling out of the colon combined with greater omentum with pedicle packing and made a success in the treatment of RVF. These results indicated that greater omentum with vascular pedicles could be effective in repairing the posterior vaginal wall injury and strengthening the posterior vaginal wall and anterior rectal wall. Next, two domestic studies [7, 8] reported to make a success on the prevention of RVF by packing the rectovaginal area with greater omentum. But in our actual practice, the greater omentum could be pulled down to the space between the rectum and vagina in some patients, which made the uncertainty of the prevention effect increase.

Inspired by preventing RVF through packing the rectum and vagina with vascular pedicled greater omentum with its shortcomings, we propose the prevention of RVF by using autologous fat flap containing ovarian vascular pedicle to pack the rectum and vagina after radiotherapy and chemotherapy in female rectal cancer patients.RVF causes great suffering to women and is still a major problem in treatment. Therefore, a new method with good effect in preventing RVF during rectal cancer radical operation is needed and to be validated by a high-quality randomized controlled trial (RCT).

### Objectives {7}

In this study, packing the anastomotic stoma with a laparoscopic dislocated fat flap containing ovarian vascular pedicle anterior to the anastomosis to explore whether a statistically significant difference in the incidence of rectovaginal leakage between the experimental group and the control group. Thus, we study the feasibility of this method for the prevention of postoperative rectovaginal fistula, and to provide effective clinical evidence for the prevention of postoperative RVF. The purpose study’s main aim is to evaluate the intervention’s efficacy while analyzing feasibility. The purpose of the study is to evaluate the effectiveness of the intervention methods (preventing rectovaginal fistula) in the experimental group, as well as the feasibility of their clinical implementation and promotion. Finally, we attempt to analyze the population suitable for using this method from the final statistical data.

### Trial design {8}

This is a single-site parallel group RCT designed to explore the feasibility of packing with laparoscopic dislocated fat flap containing ovarian vascular pedicle anterior to the anastomotic stoma after rectal cancer surgery to prevent RVF. It will be implemented in Fujian Medical University Union Hospital from April 2020 to March 2023. Female patients with low and medium rectal cancer who are eligible will be randomly assigned to the experimental group or the control group in a 1:1 allocation ratio. The sample size should achieve the goal of non-inferiority. All patients will sign the informed consent form before they are enrolled in the trial (Additional file 1).

## Methods: participants, interventions, and outcomes

### Study setting {9}

All eligible participants will be recruited through the outpatient clinic or inpatient, hospital-based WeChat advertising, and posters in the Fujian Medical University Union Hospital.

### Eligibility criteria {10}

All female patients undergoing low and medium rectal cancer surgery during the study period will be screened for eligibility. All eligible participants undergo a formal informed consent process by the research assistant and a research clinician. The interventions are delivered by the research clinicians.

#### Inclusion criteria

Patients who meet the following conditions will be included:

- Refer to the diagnostic and classification criteria for colorectal cancer of the American Joint Committee on Cancer (AJCC) and The Union for International Cancer Control (UICC) [9], seventh edition. The diagnosis of rectal cancer is confirmed with cTNM staging, T3–4NxM0.
- Female patients with ovarian failure after chemoradiotherapy or postmenopausal patients (serum FSH > 40U/L, LH > 20 IU/L, and E2 < 10–20 pg/mL).
- Received preoperative neoadjuvant chemoradiotherapy: radiotherapy regimen (long-range radiotherapy: DT50Gy/25F), chemotherapy regimen (XELOX regimen).
- Preoperative tumor distance to anal margin ≤ 7 cm.
- No other primary malignant tumor has been diagnosed.
- The clinical data are complete and prophylactic ileostomy is acceptable.
- The consent is signed.

#### Exclusion criteria

Patients who meet any of the following conditions will be excluded:

- The history of major abdominal surgery.
- Emergency surgery (perforation, bleeding, intestinal obstruction, etc.).
- Patients with severe heart, lung, liver, and kidney diseases who cannot tolerate laparoscopic surgery.
- Patients with coagulation dysfunction.
- Patients after hysterectomy and oophorectomy.

### Who will take informed consent? {26a}

After being evaluated according to the inclusion and exclusion criteria, eligible individuals will be approached for participation in the trial by a researcher with a research assistant during their preoperative visit. All participants will be clearly informed about the potential benefits and risks of the trial before they sign the informed consent.

### Additional consent provisions for collection and use of participant data and biological specimens {26b}

Not applicable. There are no plans for secondary or ancillary studies using the data generated in this trial.

## Interventions

### Explanation for the choice of comparators {6b}

This trial will compare the efficacy of preventing RVF. Compared to the control group, the experimental group would add a step to an operation of packing with a dislocated fat flap containing ovarian vascular pedicle anterior. The operations will be performed by the same clinic doctor of the trial at Fujian Medical University Union Hospital. It has the advantages of not being easily perceived by the patient and a higher feasibility of operation.

### Intervention description {11a}

#### Group A (experimental group)

**Fig. 1:**
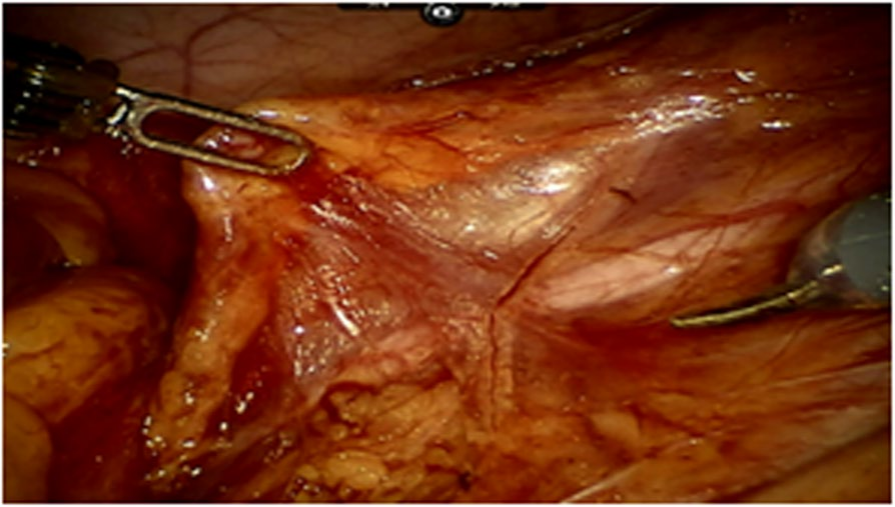
Separate the ovarian vessels and protect the ureter

**Fig. 2:**
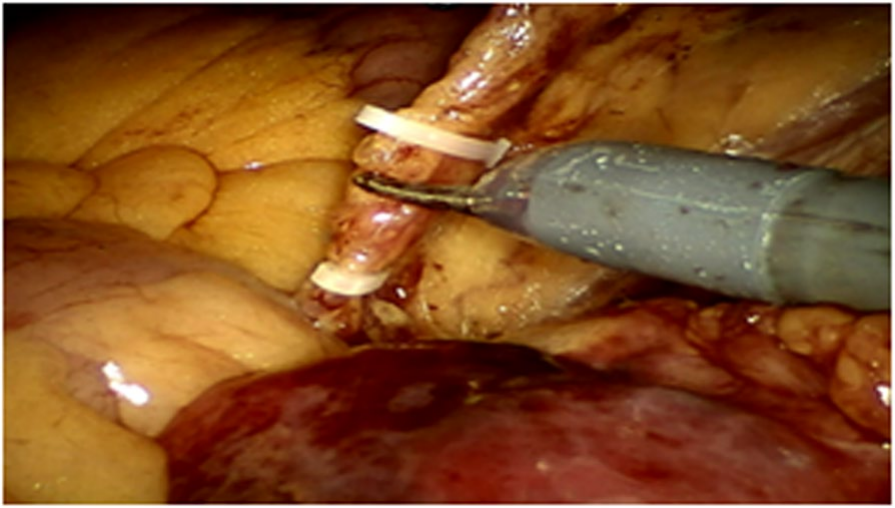
Sever the ovarian vessels

**Fig. 3:**
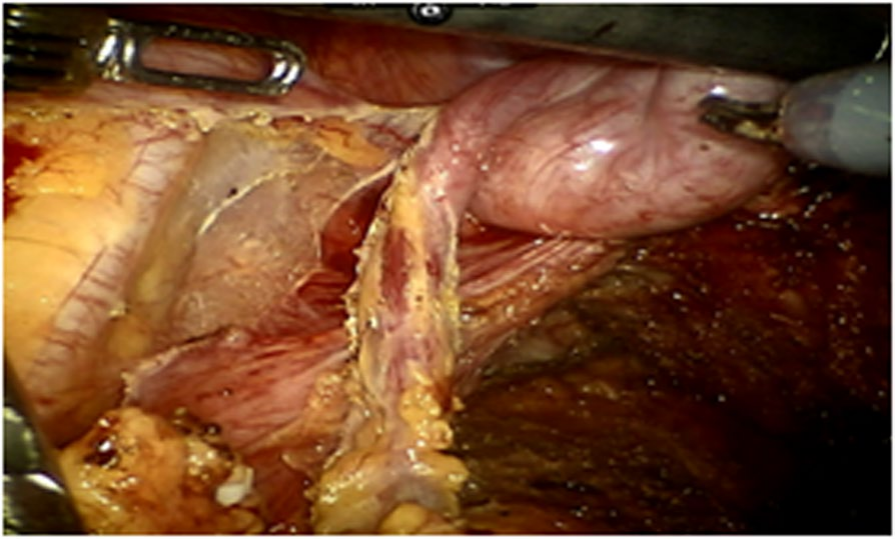
Dislocate the fat flap with 10–15 cm in total length

**Fig. 4:**
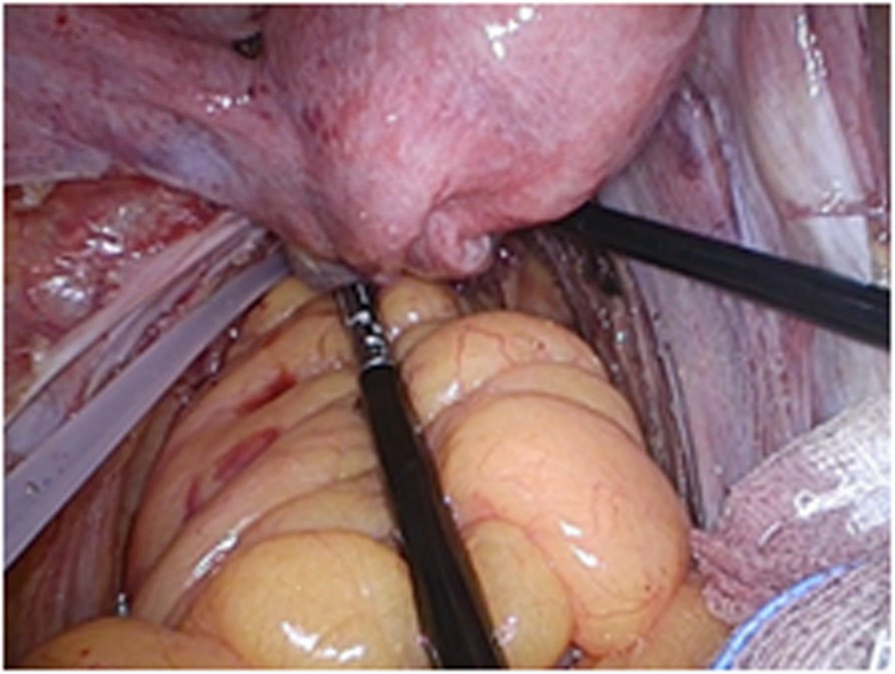
The fat flap containing ovarian vascular pedicle packed between the anastomotic stoma and vagina, then fixed with medical fibrin glue

- All operations will be performed according to the principle of TME radical resection in rectal cancer surgery. The anterior rectal wall of about 1 cm distal to the anastomosis will be dislocated.
- Before the rectal anastomosis, the researcher would expose one side of the ureter (usually the left side), dislocate the fat flap containing ovarian vascular pedicle to avoid damage of the ureter, and dissect ovarian vessels proximal to the kidney. After dislocating the fat flap, the size of the packing end would be 2 cm (width) × 4–5 cm (length) at least, with a 10–15 cm in total length, which would be measured by a thread during the operation. This length would ensure to pull-down to 5 cm below the anastomosis (Figs. 1, 2, and 3).
- After rectal anastomosis, a large amount of normal saline will be used to rinse the pelvic cavity. The liquid will be sucked up. The surface of the rectum and vagina will be dried with gauze.
- The fat flap containing ovarian vascular pedicle will be flipped downward and packed between the anastomotic stoma and vagina (9 o’clock to 3 o’clock of the outer wall of the rectum in lithotomy position), then fixed with medical fibrin glue. (Fig. 4)

#### Group B (control group)

All operations will be performed according to the principle of TME radical resection in rectal cancer surgery.

### Criteria for discontinuing or modifying allocated interventions {11b}

- The patient has complications before surgery.
- The patient agrees to the study but refuses to be randomly assigned.
- Conditions not suitable for the protocol are found during surgery. For example, during the surgical process, it is found that the patient does not have a suitable free fat flap with ovarian vascular pedicle available, or there is a need to change the surgical method or expand the surgical scope in crisis situations such as bleeding during the operation, or special circumstances with the location, size, and surrounding tissues discovered during the operation.
- The patient asks to be withdrawn from the protocol.
- The patient would be unable to continue the protocol due to other postoperative complications.
- Patients lost to follow-up.

### Strategies to improve adherence to interventions {11c}

- Explain the details of the experiment to the patient when giving informed consent. Provide participants with an adequate understanding of the risks and benefits.
- The diagnosis must be correct for the subject under study.
- The prevention and treatment measures given should be effective and without serious adverse reactions.
- Patients who accept prevention and treatment measures must adhere to voluntary rather than forced.
- Patients fully understand the purpose and significance of treatment and actively accept effective treatment.
- Improve the quality of medical services and maintain a good relationship between researchers and participants to improve patient compliance.
- All the surgeons participating in the experiment signed relevant experimental agreements, including the Non-disclosure agreement, the standardized operation agreement according to the experimental scheme, and the agreement for truthful reporting of medical records. These strategies are supervised and implemented by the Ethics Committee.

### Relevant concomitant care permitted or prohibited during the trial {11d}

- The similar type of surgery is prohibited during the trial
- Other complications arising from the procedure can be managed during the trial.
- Patients who drop out of the experiment during the course of the study will be no longer included in the analyses.
- Patients with other fatal complications will be no longer included in the analyses.
- All routine treatment procedures for women in care for low and medium rectal cancer, as deemed appropriate by the patient’s treating clinicians, will be permitted during the trial.
- Additional clinical review, further treatments, including treatment of rectovaginal fistula, and concomitant medications will be determined by clinical need and will be recorded in follow-up case report forms.

### Provisions for post-trial care {30}

At the end of the trial, participants will return to the care of their treating clinician to determine any further treatment as required.

### Outcomes {12}

#### The primary outcomes

The primary objective of this trial is to demonstrate the efficacy outcomes of the experimental group in preventing RVF. This includes monitoring the difference in incidence of RVF between the two groups.

#### Secondary outcomes

- The occurrence time of postoperative RVF.
- The occurrence time of RVF after stoma closure.
- Postoperative complications, such as anastomotic leakage, chylous leakage, and intestinal obstruction.
- Postoperative serological test indexes, such as FSH, LH, E2, CEA, and CA199.
- Others about evaluating surgical methods, such as the duration of the operation, quality of life, abnormal discharge in the vagina.

The score of a functional assessment of cancer therapy (FACT) system will be used to evaluate the patient’s quality of life.

All data about the outcome will be collected according to the follow-up timepoint in Table 1. Measurement data between the two groups will be analyzed with the *t*-test and rank-sum test, while categorical data will be analyzed with *χ*^2^ test. The duration time will be recorded as mean ± standard deviation, and the incidence data will be recorded as median (first quartile, third quartile). The significance level used for statistical analysis will be two-sided with confidence intervals at the 95% level. All results will be applied to analyze the reliability of the new operation method, and to explore whether this new operation method will cause changes in the related complications, female hormones, affect the effect of the operation, and quality of life.

**Table 1:** Schedule of enrolment, intervention, and assessments

Timepoint	Screening	Baseline	Intervention		Follow-up(months)											
			**Before**	**After**	**0**	**3**	**6**	**9**	**12**	**15**	**18**	**21**	**24**	**36**	**48**	**60**
***Basic information***																
Informed consent	x															
Inclusion/exclusion	x	x														
***Interventions***																
Group A			x	x												
Group B			x	x												
***Assessments***																
**Primary outcome**																
RVF occurrence						x	x	x	x	x	x	x	x	x	x	x
**Secondary outcomes**																
The occurrence time of postoperative RVF						x	x	x	x	x	x	x	x	x	x	x
The occurrence time of RVF after stoma closure						x	x	x	x	x	x	x	x	x	x	x
Postoperative complications																
Anastomotic leakage						x	x	x	x	x	x	x	x	x	x	x
Chylous leakage						x	x	x	x	x	x	x	x	x	x	x
Intestinal obstruction						x	x	x	x	x	x	x	x	x	x	x
Postoperative serological test indexes																
FSH		x				x	x	x	x	x	x	x	x	x	x	x
LH		x				x	x	x	x	x	x	x	x	x	x	x
E2		x				x	x	x	x	x	x	x	x	x	x	x
CEA		x				x	x	x	x	x	x	x	x	x	x	x
CA199		x				x	x	x	x	x	x	x	x	x	x	x
The duration of the operation					x											
Abnormal discharge in the vagina						x	x	x	x	x	x	x	x	x	x	x
Quality of life		x				x	x	x	x	x	x	x	x	x	x	x

*RVF* rectovaginal fistula, *FSH* follicle-stimulating hormone, *LH* luteinizing hormone, *E2* estradiol, *CEA* carcinoembryonic antigen, *CA199* carbohydrate antigen199

### Participant timeline {13}

The timing of intervention and data collection is detailed in Table 1.

### Sample size {14}

Female patients with low and medium rectal cancer in the Department of Colorectal Surgery, Fujian Medical University Union Hospital, from April 2020 to March 2023 are selected as the research participants. It is assumed that the RVF of the study group is the same as that of the control group. According to previous research results and relevant literature reports, the incidence of RVF of the control group and the test group was set at 9.9%, the non-inferior margin value was set at 15%, the test level was set at 0.05, and the power was set at 0.8 [1, 2]. The sample size was calculated by SPSS24.0 software as follows: *N* = 115, and each group needed 115 people. We assume that 20% of patients would be lost to follow-up, and 144 would need to be enrolled.

### Recruitment {15}

- Participants with stable conditions are selected, and the participants are accurately defined.
- Disease status and related variables are accurately measured.
- Investigators and clinical trial coordinators should receive training to ensure that participants are selected and excluded strictly according to protocol, and studies are conducted according to protocol.
- Recruited from a population at high risk for post-operative RVF.
- To improve the identification of participants based on the historical data and literature reports of the study group.

## Assignment of interventions: allocation

### Sequence generation {16a}

Randomization occurs after recruitment. Participants are randomized to Group A (experimental group) and Group B (control group) using a computerized random number generator in R software by a research assistant, which divides eligible participants into Group A or Group B with a 1:1 ratio.

### Concealment mechanism {16b}

Allocation will be concealed, and envelopes with participant numbers containing the group allocation are prepared and opened by different research assistants in front of the participants after formal consent is obtained. Both the research assistant and the participant do not have prior knowledge of the group allocation details.

### Implementation {16c}

An independent researcher, blinded to the study protocol, will generate the allocation sequence. Another researcher will give the opaque envelope to the surgeon according to the timing sequence of participant registration for the trial. Then, the surgeon will open the envelope to perform the corresponding operation.

## Assignment of interventions: blinding

### Who will be blinded {17a}

Patients are not blinded. Caregivers are not blinded. Statisticians will not know the allocation.

### Procedure for unblinding if needed {17b}

Not applicable. Because the researcher who is responsible for participant intervention will not turn a blind eye to the group assignment. Statisticians can be notified to delete data related to participants with a specific participant number.

## Data collection and management

### Plans for assessment and collection of outcomes {18a}

Case report forms (CRFs) will record all required information with separate CRFs collecting clinical and patient-reported information. Copies of CRFs used in the trial are available on request.

### Plans to promote participant retention and complete follow-up {18b}

The research assistant will contact participants via telephone call (or other contact means such as emails or text messages) to complete the later stages of the follow-up questionnaires. The research assistant also will monitor the retention rate and reasons for discontinuation of the study (e.g., consent withdrawn, lost to follow-up).

### Data management {19}

Researchers must ensure that the privacy of participants. In all documents, only the identity of the clinical study subject should be identified, and the name and hospitalization number of the subject should not be indicated. The investigator must maintain the names, addresses, and records of all participants involved in the clinical trial. The raw data will be collected by assessors who are blinded to the group assignment and repeated input methods will be used to ensure that the entered data is correct. These data will be kept strictly by the researchers.

### Confidentiality {27}

This study intends to sign a clinical trial informed consent prior to surgery to inform patients of their relevant rights and interests. Patients’ data included in this study will be confidential, patient specimens will be identified by study numbers rather than patient names, and patient information will not be disclosed to members outside the study team. No patient information will be disclosed when the study results are published. All patients in the experimental group have ovarian failure, and the study would not increase the additional risk and cost of patients, while the control group only has radical rectal cancer surgery, which would not increase the additional risk and cost of patients. We will protect the privacy of participants’ personal medical information as required by law. The staff in this study is also bound by this agreement.

### Plans for collection, laboratory evaluation, and storage of biological specimens for genetic or molecular analysis in this trial/future use {33}

Not applicable. This trial does not have biological specimens.

## Statistical methods

### Statistical methods for primary and secondary outcomes {20a}

The independent statistician is responsible for the statistical analysis with SPSS 24.0 software. Patient data that falls off will not be included in the analysis. In the statistical analysis, measurement data between the two groups will be analyzed with the *t*-test and rank-sum test, while categorical data will be analyzed with *χ*^2^ test. Data will be recorded as mean ± standard deviation or median (first quartile, third quartile). The experiment is a non-inferiority trial, and the statistical method is mainly based on “parameter estimation.” Using the primary endpoint, determine the confidence interval for the difference in the efficacy of surgical methods in preventing rectovaginal fistula between the control group and the cases group. The significance level used for statistical analysis will be two-sided with confidence intervals at the 95% level.

### Interim analyses {21b}

#### Objective

To comprehensively weigh the cost-risk relationship of this surgical method in preventing rectovaginal fistula from the perspective of the effectiveness and safety of the plan in the interim. To confirm whether the intervention can reach the study endpoint.

#### Samples

It is expected that the total number of samples would reach 80–100. The cases and controls were grouped according to the experimental protocol, and mid-term experimental data would be collected to analyze the expected risks of intervention measures. When adverse events or other related complications occur, patients who consider adverse outcomes would be reported by the research team and analyzed to determine whether their adverse events are related to the trial, and the research team would fulfill their obligation to inform and respect the patient's right to know related content. In the case of mid-term analysis, if there is no statistically significant difference between the case group and the control group or there are more adverse complications in the case group compared to the control group, the trial can be considered for discontinuation and protocol modification can be carried out. If there are significant modifications during the implementation process, the main researcher shall write and sign a “protocol modification manual” and submit it to the ethics committee for approval before implementation.

In the study, if the analysis results have no clinical significance due to the number of cases, the experimental time will be extended. The interim analysis report will be publicly released by the research participants.

### Methods for additional analyses (e.g., subgroup analyses) {20b}

Not applicable. No subgroup analyses are planned. Significant interaction effects will be used to suggest a differential effect of treatment across different patient groups.

### Methods in analysis to handle protocol non-adherence and any statistical methods to handle missing data {20c}

We perform statistical analysis on the complete case. The complete case is that all the data required for this study protocol are complete and there are no missing cases in order to be included in the final statistical analysis. The premise of this RCT is that it does not violate the treatment principles of the patient's disease and does not imply or modify the patient's treatment intention. The experiment respects the treatment willingness of each enrolled patient.

### Plans to give access to the full protocol, participant-level data, and statistical code {31c}

The original data will be published and shared in the Chinese Clinical Trial Registry (ChiCTR, https://www.chictr.org.cn/searchproj.html). And accept the supervision of the center.

## Oversight and monitoring

### Composition of the coordinating center and trial steering committee {5d}

In order to control the quality of the clinical trial, the whole process of the trial will be conducted under the supervision of a qualified clinical trial expert at Fujian Medical University Union Hospital. The qualified clinical trial expert of Fujian Medical University Union Hospital will conduct the data monitoring. When problems occur in the trial, the expert has the right to make the final decision to terminate the trial if necessary. Any changes will be notified in writing to all participants in the trial after approval by the ethics committee. The principle investigator will be fully responsible for conducting the trial and will make any final decisions.

### Composition of the data monitoring committee, its role and reporting structure {21a}

In order to control the quality of the clinical trial, the whole process of the trial will be conducted under the supervision of a qualified clinical trial expert at Fujian Medical University Union Hospital. The qualified clinical trial expert of Fujian Medical University Union Hospital will conduct the data monitoring. When problems occur in the trial, the expert has the right to make the final decision to terminate the trial if necessary. Any changes will be notified in writing to all participants in the trial after approval by the ethics committee. The principle investigator will be fully responsible for conducting the trial and will make any final decisions.

### Adverse event reporting and harms {22}

Risks greater than routine operation incurred through participating in this trial are not anticipated. As a result, no discomfort or adverse events are anticipated for this study. However, in the study debrief form, participants will be provided with routes for providing feedback for seeking support. Specifically, participants will read the following text: “If you have any issues that have arisen through participating in this study, you may wish to contact the research assistant and obtain a clinical management plan from the clinician.” All adverse events (reported and observed) will be reported to the Research Ethics Committee and recorded by the research team, with respect to any of the following types of harm occurring in the study, and the likelihood, severity, and consequence of those harms occurring.

### Frequency and plans for auditing trial conduct {23}

We will audit according to time points in Table 1; any modification that may have an impact on the study and potential benefit to patients or affect patient safety, including changes in the study objective, study design, patient population, sample size, study procedure, or serious adverse events, will be reported to the Committee. This will be decided jointly with the monitoring committee, and approved by the Ethics Committee.

### Plans for communicating important protocol amendments to relevant parties (e.g., trial participants, ethical committees) {25}

Changes to the proposed protocol will require a resubmission to the China Clinical Trial Registry and re-approved by the Ethics Committee. If changes to the protocol are different from what was explained to participants during consent, participants will be duly informed of the modifications and re-consented by research staff before continuing in the study.

### Dissemination plans {31a}

The trial results will be disseminated to the public and to relevant clinical and academic communities. This will be done through published papers, conference presentations, university classes, etc.

## Discussion

With the promotion of anus preservation surgery for low rectal cancer, the occurrence of RVF after rectal cancer in women has been increasing recently. The incidence of RVF after anterior resection of rectal cancer in women is not low, which has been reported to be 0.9–9.9% in most literatures [1, 2]. Most of the existing studies focus on the treatment of RVF, but relatively few studies concentrate on prevention. With the research on postoperative RVF of low-middle rectal cancer, results have shown that intraoperative measures can effectively reduce the incidence of RVF in terms of surgical techniques [4]. For instance, push and pull the uterine and vaginal walls upward and outward which can make a certain tension with the anterior rectal wall; control the frequency of electric knife or ultrasonic knife; examine the vaginal wall via pelvis and vagina prior anastomat triggering to avoid being clamped; block the vaginal wall with pressure intestinal spatula if necessary, etc. Gu et al. proposed that proactive interventions should be taken for patients with high risk factors of RVF [10]. For patients who are not satisfied with combined partial vaginal posterior wall resection or vaginal posterior wall separation, prophylactically packing of greater omentum or levator ani muscle flap in rectovaginal space can be considered to increase local blood supply and reduce local inflammation.

In the treatment of RVF, scholars [5, 6] had tried trancanal pulling out of the colon combined with greater omentum with pedicle packing and made a success. These results suggest that greater omentum with vascular pedicles may be effective in repairing the posterior vaginal wall injury and strengthening the posterior vaginal wall and anterior rectal wall. Therefore, inspired by this, two domestic studies [7, 8] have achieved good results on the prevention of RVF after rectal cancer by packing the rectovaginal area with greater omentum. However, these two studies have shortcomings. First, the study only investigated the risk of RVF and other complications, and its results did not fully reflect the recovery of the posterior vaginal wall. Second, limited patients were included in these two studies, which were 58 cases in one study [7] and 46 cases in the other [8]. Third, this study showed that the operation time of the experimental group (greater omentum packing group) was significantly longer than that of the control group, and it was statistically significant [7]. In addition, in our actual practice, only a partial of the patient’s omentum was able to be pulled downwards to the space between the rectum and vagina. The uncertainty of preventive effect was increased.

Inspired by the approach of preventing RVF through packing the rectum and vagina with vascular pedicled greater omentum with its shortcomings, we introduce the prevention of RVF by using an autologous fat flap containing ovarian vascular pedicle to pack the rectum and vagina after radiotherapy and chemotherapy in female rectal cancer patients. 39 cases of packing with fat flap containing ovarian vascular pedicle anterior to the anastomotic stoma were performed. After the follow-up period of 3–14 months, no RVF was observed. The mechanism of this approach in preventing RVF depends on the following three aspects: First, the adequate blood supply of the packing tissue should be ensured. Both the greater omentum containing vascular pedicle and ovarian fat flap are autologous and living tissues with blood vessels. The ovarian blood supply comes from the uterine artery and the ovarian artery, so the ovarian and its surrounding tissues are rich in blood supply, ensuring the activity of the tissue packing between the rectum and vagina. Second, the fat flap with ovarian vascular pedicle needs to be tensionless and can be smoothly fitted to the rectal anastomosis and the posterior wall of the vagina. The length of the dislocated fat flap with the ovarian vascular pedicle is measured during the operation. The fat flap, which is not found, could not be pulled down to the pelvic floor due to its insufficient length, making up for the deficiency of greater omentum packing. Finally, we tried various methods of packing fixation. Initially, Hem-o-lok was used to clamp the distal stump of the ovarian vessels, and then it was fixed between the rectal anastomosis and the vagina. However, during postoperative follow-up, we found that Hem-o-lok could be palpated by the anterior rectal wall or posterior vaginal wall during physical examination, which may affect the quality of sexual intercourse. We improved this procedure by ligating the distal stump of the ovarian vessels with absorbable thread. Moreover, we fixed it with chemical glue at the beginning, once the glue spills in the pelvic cavity by maloperation, it can cause adhesion of the abdominal and pelvic organs with serious consequences. Chemical glue is a foreign body, which may cause an uncontrollable local infection. Therefore, we used biological fibrin glue for fixation instead of chemical glue. Medical biological fibrin glue [11] prevents adhesion and can be dissolved by its own fibrinoclase in about 15 days after excessive use to avoid the situation mentioned above.

The mechanism of packing with a fat flap containing ovarian vascular pedicle anterior to the anastomotic stoma is similar to that of greater omentum packing. The peripheral vessels of fat flap containing ovarian vascular pedicle may adhere to inflammatory tissue in the posterior wall of the vagina and form collateral circulation. The local blood flow of the posterior vaginal wall may be improved, providing rich nutrition and a good healing environment for the posterior vaginal wall. At the same time, ovarian vascular pedicle also strengthens the posterior wall of the vagina and the anterior wall of the rectum. All the above have played an important role in the prevention of RVF.

## Trial status

The trial is approved by the Chinese Clinical Trial Registry(ChiCTR) with trial identifier ChiCTR2000031449, with registration date 26.06.2019, Protocol version 1.0, date: 01.06.2019. All items of the WHO Trial registration data set can be found within the protocol.The trial is approved by the ethical reviewing board of Fujian Medical University Union Hospital(2020YF003-1), date: 02.03.2020. The recruitment for this study started in April 2020 to March 2023. We have recruited 96 people so far. The interventions have also commenced. We are currently at the beginning stages of year 2. Recruitment is expected to be completed at the end of 2023. If there are insufficient recruits during the study period, it is possible to extend the trial period.

### Supplementary Information

**Additional file 1.**

**Additional file 2.**

## Data Availability

Researchers participating in the experiment can apply for access to the data upon reasonable request and without involving disclosure. Data will be available from the corresponding author on reasonable request. Please contact xhhy@fjmu.edu.cn.

## Abbreviations

RVF: Rectovaginal fistula
TME: Total mesorectal excision
RCT: Randomized controlled trial
AJCC: American Joint Committee on Cancer
UICC: Union for International Cancer Control
CRC: Colorectal cancer
CRFs: Case report forms
FSH: Follicle-stimulating hormone
LH: Luteinizing hormone
E2: Estradiol
CEA: Carcinoembryonic antigen
CA199: Carbohydrate antigen199
