# Supplementary material for: The prevention of rectovaginal fistula after rectal cancer surgery by packing with laparoscopic dislocated fat flap containing ovarian vascular pedicle anterior to the anastomotic stoma: a parallel group randomized controlled trial protocol
Source: Trials. 2024 Jan 18;25:63. doi: 10.1186/s13063-023-07721-2 (PMC10795231; doi:10.1186/s13063-023-07721-2)
Supplement: Supplementary file 2 — Additional file 2. [file 13063_2023_7721_MOESM2_ESM.docx]

**应用带卵巢血管蒂脂肪瓣填塞直肠吻合口前方预防低位直肠癌术后直肠阴道瘘的前瞻性可行性研究**

**知情同意书**

**版本：v.1.0**

**日期：2019.06.01**

亲爱的患者：

感谢您参与本研究，本研究采用单中心、随机对照实验设计。本研究拟通过对新辅助放化疗后中低位直肠癌女性患者在腔镜手术中利用游离带卵巢血管蒂脂肪瓣填塞直肠阴道间隙，研究该方法预防术后直肠阴道瘘的可行性。本研究不涉及肿瘤根治手术效果，将不会影响患者的长期预后。

如果您同意参与这项研究，我们将和您或您的家人进行详细沟通，向您介绍该项研究的有关情况，也请您提供与疾病有关的情况，包括发病过程、家族史、以前就诊情况及曾经做过一些检查结果等。我们将对每位参与者进行编号，建立研究档案。

一、研究背景和研究目的

本研究拟通过对放化疗后中低位直肠癌女性患者在腔镜手术中利用游离带卵巢血管蒂脂肪瓣填塞直肠阴道间隙，研究该方法预防术后直肠阴道瘘的可行性。以期为临床上预防术后RVF提供有效的临床依据。

二、哪些人不宜参加研究

本研究有严格的纳入标准和排除标准，凡不符合纳入标准的患者均不宜参加本研究，另外还有研究人员认为其他原因不适合临床研究者。

三、如果参加研究将需要做什么？

1. 在您入选研究前，医护人员将询问、记录您的病史。您是合格的纳入者，您自愿参加研究，签署知情同意书。如您不愿意参加研究，我们将按您的意愿处理。

1. 若您自愿参加研究，将按以下步骤进行：本研究采用单中心、随机对照的观察性实验设计，分为实验组和对照组。
2. **实验组：**按直肠癌手术D3根治原则进行手术操作，同时需保证吻合口远端约1 cm的直肠前壁是游离的，完成直肠吻合后，显露一侧输尿管，游离该侧带卵巢血管蒂脂肪瓣，长约10-15cm，避免损伤输尿管，离断卵巢血管近肾侧，大量生理盐水冲洗盆腔，吸尽液体，纱布擦干直肠与阴道两面，将带卵巢血管蒂脂肪瓣翻下填塞在直肠吻合口与阴道之间（截石位直肠外壁9点至3点之间），生物蛋白胶水固定。
3. **对照组：**直肠癌手术D3根治术进行手术操作，其余无特殊。对比分析在低位直肠癌手术中利用游离带卵巢血管蒂脂肪瓣填塞直肠阴道间隙，来预防术后直肠阴道瘘的可行性。

四、参加研究可能的受益

如果您参加了该研究，在您今后的结直肠癌治疗、康复过程中，将获得以下更为完善的诊治、随访及康复的支持。包括：1.更加完善的诊疗措施，由专家进行临床讨论进行更缜密的治疗方案，使者得到最好的诊疗。2.更加完善随访监测计划：本研究将设立专门的研究委员会，对您开展定期的随访计划提醒及实施，让您的术后健康状况得到全方位的监测及最佳医疗措施支持。3.专门的随访、复査门诊与咨询：本研究将将设立随访、复查门诊，让您得到及时、全面的术后病情咨询与监测，并对让您在术后的康复、生活中有关结肠癌的相关问题进行及时的回答或处理。此外，该研究结果将对所有该疾病患者人群的临床决策具有重要的意义，并可为将来的临床实践提供指导。

五、参加研究可能的风险、不良反应和不适、不方便

本项目手术方式、相关检查均为目前临床常规开展的手术及检查方法，因此参加本项目本身不会增加您的风险。您在研究期间需要按时到医院随访，做一些必要的检查，这些将占用您一些时间，也可能给您造成麻烦或带来不方便。 临床治疗后，包括在研究期间如果您出现任何不适，或者病情出现新的变化，或任何意外情况，不管是否与研究有关，均应及时通知您的医生，医生将对此做出判断并给予适当的医疗处理。

六、有关费用

由于本研究所涉及的方案均是我科结肠癌治疗的常规所用方法，并未额外增加手术风险及增加手术费用。对于您同时合并的其他疾病所需要的治疗和检查，将不在免费的范围以内。

七、个人信息是保密的吗？

您的医疗记录将完整地保存在您所就诊的医院。医生会将化验及其他检查结果记录在您的病历上。研究者、伦理委员会和药品监督管理部门将被允许查阅您的医疗记录。记录您所有的个人信息，包括姓名、电话、电子邮件、住址等均不会出现在电子数据库中，任何有关本研究结果的公开报告将不会披露您的个人身份及信息。我们将在法律允许的范围内，尽一切 努力保护您的个人医疗资料的隐私。

八、怎样获得更多的信息？

您可以在任何时间提出有关本研究的任何问题，并得到相应的解答。如果在研究过程中任何重要的新信息，可能影响您继续参加研究的意愿时，您的医生将会及时通知您。

九、可自愿选择参加和退出研究

是否参加研究完全取决于您的意愿。您可以拒绝参加此项研究，或在研究过程中的任何时间退出本研究，这都不会影响您和医生间的关系，都不会影响对您的医疗或有其他方面利益的损失。

出于对您的最大利益考虑，医生或研究者可能在研究过程中随时中止您继续参加本研究。 如果您因为任何原因从本研究中退出，如果医生认为临床需要，您也可能被要求进行实验室检查和体格检查。

十、现在该做什么？

是否参加本项研究由您自己（和您家人）决定。 在您做出参加研究决定前，请尽可能向您的医生询问有关问题。 感谢您阅读以上材料。如果您决定参加本项目，请告诉您的医生，他/她会为您安排一切有关研究的事务。请您保留这份资料。

同意声明

我已经阅读了上述有关本研究的介绍，并且有机会就此项研究与医生讨论并提出有关问题。

我提出的所有问题都得到了满意的答复。 我知道参加本研究可能产生的风险和受益。我知晓参加本研究是自愿的，我确认已有充足的时间对此进行考虑，而且明白：

 我可以随时向医生咨询更多的信息，联系电话：0591-86218010。

 我可以随时退出本研究，而不会受到歧视或报复，医疗待遇与权益不会受到任何影响。

我同样清楚，如果我中途退出研究，我若将我的病情变化告诉医生，完成相应的体格检查和理化检查，这将对整个研究十分有利。 如果因病情变化我需要采取任何其他药物治疗，我会在事先征求医生的意见，或在事后如实告诉医生。 我同意药品监督管理部门、伦理委员会或申办者代表查阅我的研究资料。 我将获得一份经过签名并注明日期的知情同意书副本。 最后，我决定同意参加本项研究，并保证尽量遵从医嘱。

患者姓名（正楷）： 患者签字： 日期：

身份证号码：

如患者指定过合法委托人（如适用，并签署委托协议书）

被委托人姓名（正楷）： 委托人签字： 日期：

身份证号码： 与委托人关系：

我确定已向患者解释了本研究的详细情况，包括其权力以及可能的受益和风险，并给其一份签署过的知情同意书副本。

研究者姓名（正楷）： 签字： 日期：
